# Impact of timing of a second colostrum feeding on serum immunoglobulin G dynamics in calves during the first week of life

**DOI:** 10.3168/jdsc.2025-0870

**Published:** 2025-11-13

**Authors:** Maximilian A.E. von Riedheim, Hannes Erkinger, Josef J. Gross

**Affiliations:** Veterinary Physiology, Vetsuisse Faculty, University of Bern, Bremgartenstrasse 109a, 3012 Bern, Switzerland

## Abstract

This study evaluated whether a high-IgG colostrum replacer administered at 24 h postpartum (p.p.) influences IgG absorption in neonatal calves. Twelve Holstein and crossbred calves were assigned to 2 groups: CCT (n = 6) received 2.5 L of colostrum replacer (100 mg IgG/mL) at 4 and 12 h p.p., followed by enriched bulk tank milk at 24 h p.p.; CTC (n = 6) received the same colostrum replacer at 4 and 24 h p.p., with enriched bulk tank milk at 12 h p.p. Enriched bulk tank milk was bulk tank milk supplemented with 27 g/L colostrum replacer, mimicking transition feeding. Serum IgG, total protein, γ-glutamyl transferase, and apparent efficiency of absorption (AEA) were measured. The IgG concentrations and AEA were compared at 24 h p.p. in CCT and on d 2 (32–38 h p.p.) in CTC to reflect the status after the second colostrum feeding in each group. At these time points, CTC calves had lower IgG (16.9 ± 1.1 vs. 23.3 ± 1.8 mg/mL) and AEA (11.7% vs. 16.8%), although IgG persisted longer. These findings clearly highlight the superiority of early colostrum feeding for achieving adequate passive immunity, while also revealing that lower initial IgG concentrations were accompanied by a relatively greater persistence during the first week of life.

## Highlights

• Early colostrum feeding is crucial for successful passive transfer of immunity.
• A second colostrum feeding at 24 hours p.p. increased serum IgG by only 4%.
• Lower serum IgG was linked to longer apparent persistence of IgG.

## Graphical Abstract

**Figure:**
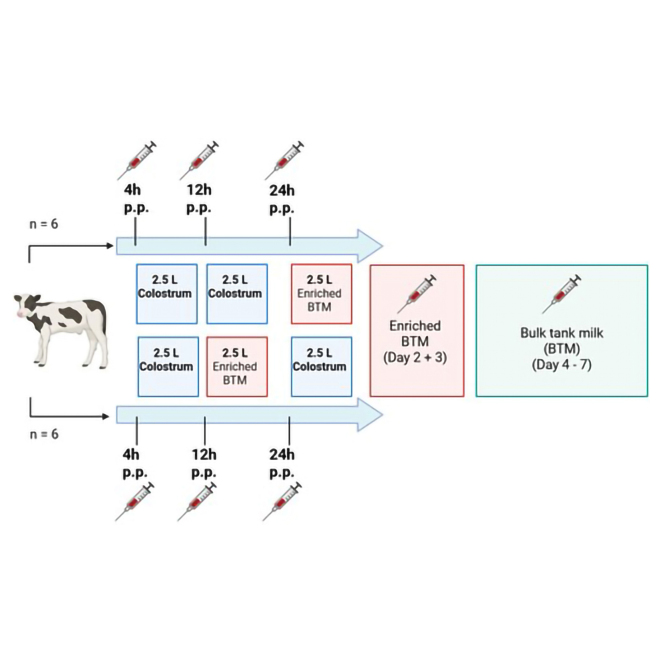
**Summary:** In this study, all calves received the same high-quality colostrum at birth, followed by either an early second colostrum feeding at 12 or 24 hours postpartum (p.p.). Although serum ?-glutamyl transferase concentrations did not differ between groups, calves receiving the second feeding at 12 hours p.p. reached higher serum immunoglobulin G (IgG) and total protein concentrations throughout the first week of life. These calves also maintained higher circulating IgG levels over time, despite the physiological post-peak decline. In contrast, calves fed the second colostrum meal at 24 hours p.p. showed a greater apparent IgG persistence relative to their lower peak concentrations. Clinical variables and growth were not affected by treatment. Overall, advancing the timing of the second colostrum feeding improves early IgG status, whereas later feeding may result in marginally more persistent IgG profiles. BTM = bulk tank milk. Created in BioRender. Freiherr von Riedheim, M. (2025) https://BioRender.com/o9kf11e.

Despite the well-known importance of colostrum for neonatal calves, field studies indicate that a substantial number of calves do not receive adequate colostrum in terms of quantity or quality. According to Beam et al. (2009), approximately 19.2% of US dairy heifer calves fail to achieve adequate passive transfer of immunity. Michelsen et al. (2025) reported that more than 29% of calves suffer from failure of transfer of passive immunity (**FTPI**). Reasons for this are multifactorial and include—among others—the broad variation in colostrum quality between individual dams (Larson et al., 1980; Quigley et al., 1994; Morin et al., 2010), as well as lacking colostrum intake by calves, particularly when left with the dam without supervision (Moran, 2012). Consequently, the immunological status of calves is often insufficient or unknown under practical farming conditions. A key physiological factor influencing colostrum efficacy is the time-limited ability of the neonatal intestine to absorb macromolecules such as immunoglobulins (Bush and Staley, 1980). This absorptive capacity declines rapidly after birth due to a process referred to as gut closure (Lecce and Morgan, 1962). Several studies indicate that absorption of IgG begins to decrease as early as 12 h postpartum (**p.p.**), with the process of gut closure likely complete by 24 h after birth (Stott et al., 1979; Bush and Staley, 1980). However, Weaver et al. (2000) and Hare et al. (2020) suggested that this closure may last up to around 36 h p.p. Moreover, evidence exists that not only timing but also IgG concentration in colostrum influences the efficiency of IgG absorption in the neonatal gut. Todd and Whyte (1995) suggested that higher concentrations of immunoglobulins may partially compensate for declining intestinal permeability, which may allow an additional passive transfer of IgG even at later time points p.p. It is well established that the gold standard for achieving successful transfer of passive immunity is to provide adequate volumes of high-quality colostrum within the first hours after birth, ideally in 2 feedings within the first 12 h (Weaver et al., 2000; Godden et al., 2019). Nevertheless, under on-farm conditions, a timely second feeding is not always feasible due to limited colostrum availability, variable quality, or management constraints. In such cases, the question arises whether a delayed second colostrum feeding, even as late as 24 h p.p., can still provide immunological benefits to the calf. Based on this rationale, the objective of the present study was to evaluate whether administration of a highly concentrated colostrum replacer at 24 h p.p. influences IgG absorption dynamics and affects the immune status of neonatal calves during the first week of life.

The present study was conducted in accordance with the Swiss law on animal welfare and was approved by the commission of animal experimentation of the canton of Fribourg (Switzerland, approval number 2024–23-FR). Twelve neonatal calves (6 female, 6 male), consisting of 8 Holstein and 4 Holstein × Limousin crossbreds, were enrolled in this study conducted at the Agroscope research station Posieux (Switzerland). Absence of dystocia, no obstetrics, and singleton birth were prerequisites for inclusion in the trial. Calving took place between January and March 2025. Immediately after birth, calves were separated from their dams to prevent colostrum ingestion, weighed, and had their umbilical cords disinfected with iodine. Calves were housed individually in straw-bedded calf hutches. Throughout the study period, calves had free access to hay and water.

After birth, calves were allocated to one of 2 experimental groups based on sex, breed, and birth weight to achieve balanced and comparable groups. This allocation was not random, which represents a limitation of the study. Group **CCT** (n = 6; birth weight 39.8 ± 1.4 kg, mean ± SEM) received a colostrum replacer (C) at 4 and 12 h after birth, as well as enriched bulk tank milk (T), prepared by adding a small amount of colostrum replacer to bulk tank milk, mimicking transition feeding, at 24 h p.p. This group included 4 Holstein calves and 2 Limousin × Holstein crossbreed calves. Group **CTC** (n = 6; birth weight 39.7 ± 1.6 kg) received colostrum replacer at 4 h p.p., the enriched bulk tank milk at 12 h p.p., and colostrum replacer again at 24 h p.p.; this group included 5 Holstein calves and 1 Limousin × Holstein crossbreed calf. The fourth meal after birth, provided at 32 to 38 h p.p. (d 2), was again enriched bulk tank milk fed in both groups. Thereafter, both groups were subjected to the same feeding regimen and were fed twice daily. On d 2 and 3, all calves received enriched bulk tank milk, providing not only additional immunoglobulins but also bioactive compounds and nutrients important for gastrointestinal development and neonatal immune maturation (Van Soest et al., 2022). From d 4 to 7, all calves received bulk tank milk twice a day, supplemented with additional carbohydrates (primarily lactose and maltodextrin, 40 g/L milk, Multi-Lac Aufzucht, Multiforsa, Switzerland). During the first 24 h, each meal consisted of 2.5 L. From d 2 onward, the volume was increased to 3.0 L per meal. Milk intake was recorded daily for each calf.

The colostrum replacer (Immune Milk, Phytobiotics Futterzusatzstoffe GmbH, Eltville, Germany) was provided in powdered form and prepared according to the manufacturer's instructions. Colostrum replacer meals were prepared by reconstituting 937g of colostrum replacer in 1.6 L of water to receive a final volume of 2.5 L. Transition milk meals were prepared by supplementing bulk tank milk with 27 g of colostrum replacer powder per liter of milk, resulting in a total of approximately 67.5 g of powder per 2.5 L feeding. On a DM basis (95.68% DM), the colostrum replacer contained 23% fat, 56% protein, 19% carbohydrates, and 280 g/kg IgG. Calves received their first 3 meals via nipple bottle, followed by nipple bucket feeding for all subsequent meals.

A standardized neonatal care protocol was implemented: General health status was monitored daily in the morning before feeding by recording heart rate, respiratory rate, and rectal temperature. In addition, fecal and health scores were determined daily using established scoring systems (Kertz and Chester-Jones, 2004; McGuirk, 2008; McGuirk and Peek, 2014).

The fat and protein content of colostrum replacer, enriched bulk tank milk, and bulk tank milk was determined by mid-infrared spectroscopy (Suisselab AG, Zollikofen, Switzerland). Carbohydrate concentrations were determined by mid-infrared spectroscopy (Suisselab AG, Switzerland). This measurement primarily reflects lactose but also includes added carbohydrates. The prepared colostrum replacer (reconstituted according to the manufacturer's instructions) contained 8.3% fat, 20.0% protein, 6.8% carbohydrates, and 100.7 mg/mL IgG, whereas the corresponding values in the enriched bulk tank milk were 5.1%, 5.4%, 5.3%, and 7.8 mg/mL, respectively. The supplemented bulk tank milk contained 4.5% fat, 4.1% protein, and 6.9% carbohydrates. The IgG concentration of bulk tank milk was 0.4 mg/mL.

Blood samples (9 mL; Vacuette, Greiner Bio-One GmbH) were collected from a jugular vein using K3 EDTA tubes for plasma (cat. no. 455036, Vacuette K3-EDTA, Greiner Bio-One) and clot activator (CAT) tubes for serum separation (cat. no. 455092, Vacuette CAT, Greiner Bio-One). The first blood sample was obtained at 4 h p.p. before colostrum intake. Subsequent samples were collected at 12 and 24 h p.p., and once on d 2 through 5 and d 7 in the morning before feeding. Subsequent samples were collected at 12 and 24 h p.p., and once on d 2 through 5 and d 7 in the morning before feeding. Day 2 corresponded to the interval from 32 to 38 h p.p., where all calves aligned into a standardized feeding and sampling schedule. Following collection, EDTA samples were placed on ice and centrifuged immediately, whereas serum samples were allowed to clot at room temperature for 2 h before centrifugation. All samples were centrifuged at 4°C for 15 min at 1,500 × *g*. Harvested plasma and serum aliquots were transferred into 1.5-mL snap-vial tubes (REF 72.690.001, Sarstedt AG & Co. KG, Nümbrecht, Germany) and stored at −80°C until analysis. Serum concentrations of total protein (**TP**, ref. 08058652190) and activity of γ-glutamyl transferase (**GGT**, ref. 08058652190) were determined enzymatically using an automated analyzer (Cobas Pure c303, Roche, Switzerland). Serum IgG concentrations were quantified using an ELISA (Bethyl Laboratories Inc.) with bovine reference serum (RS10–103), sheep anti-bovine IgG antibody (A10–118A), HRP-conjugated secondary antibody (A10–118P), TMB peroxidase substrate (SeraCare, 510–0082), and TMB BlueStop solution (SeraCare, 5150–0022). The apparent efficiency of absorption (**AEA**) of IgG was calculated as described by Quigley and Drewry (1998) using the following formula: AEA (%) = [(serum IgG concentration × plasma volume)/IgG intake] × 100. Plasma volume was estimated as 9.0% of birth BW (Quigley et al., 2002). Intake of IgG was determined by multiplying the IgG concentration in colostrum by the respective volume fed (Quigley and Drewry, 1998).

Statistical analysis was performed using SAS (version 9.4; SAS Institute Inc., Cary, NC). Data presented in this article are mean values ± SEM. To assess differences between groups over time, a mixed model was used (MIXED procedure). Fixed effects included group, day, and the group × day interaction, whereas the individual calf was treated as a repeated measure. The Akaike information criterion (lower corrected Akaike information criterion) and Bayesian information criterion were used to evaluate model selection. Significance was assumed at *P* < 0.05, whereas *P*-values between 0.05 and 0.10 indicated tendencies toward significance.

From d 1 to 7 of the study, calves in both groups gained in BW (*P* < 0.05; data not shown). No significant group effect was observed for BW. Rectal temperature increased in both groups from d 1 to 3 (*P* < 0.001; data not shown) and remained constant through d 7. No group effect was observed for rectal temperature (*P* = 0.65). Baseline serum IgG concentrations were 0 mg/mL in both groups before the first feeding, as all calves were separated from their dams immediately after birth and no significant differences were observed between groups (*P* = 0.93). Serum IgG concentrations increased significantly in both groups at 12 h p.p. following the first colostrum feeding (*P* < 0.05). In CCT, a further significant increase up to 23.3 ± 1.8 mg/mL was observed from 12 to 24 h p.p. following the second colostrum feeding (*P* < 0.01). In CTC, IgG concentrations tended to increase from 12 h to 2 d after receiving one enriched bulk tank milk meal (*P* < 0.10). Immunoglobulin G concentration was highest on d 2 in CTC (16.9 ± 1.1 mg/mL). Colostrum replacer feeding in CTC at 24 h p.p. did not further increase circulating IgG concentration. From 24 h onward, IgG concentration was greater in CCT compared with CTC (*P* < 0.05, Figure 1A). After reaching peak values, IgG concentrations declined in both groups until d 7 p.p. (*P* < 0.01). The decline in IgG concentration during the first week of life was more pronounced in CCT compared with CTC (*P* < 0.01, Figure 1B). The AEA did not differ between groups at 12 h p.p. (*P* > 0.05; Table 1), whereas at 24 h p.p., AEA was higher in CCT compared with CTC (22.0 ± 1.5% vs. 16.8 ± 1.3%; *P* < 0.05; Table 1). On d 2, AEA did not differ between groups (*P* > 0.05; Table 1).

**Figure 1:**
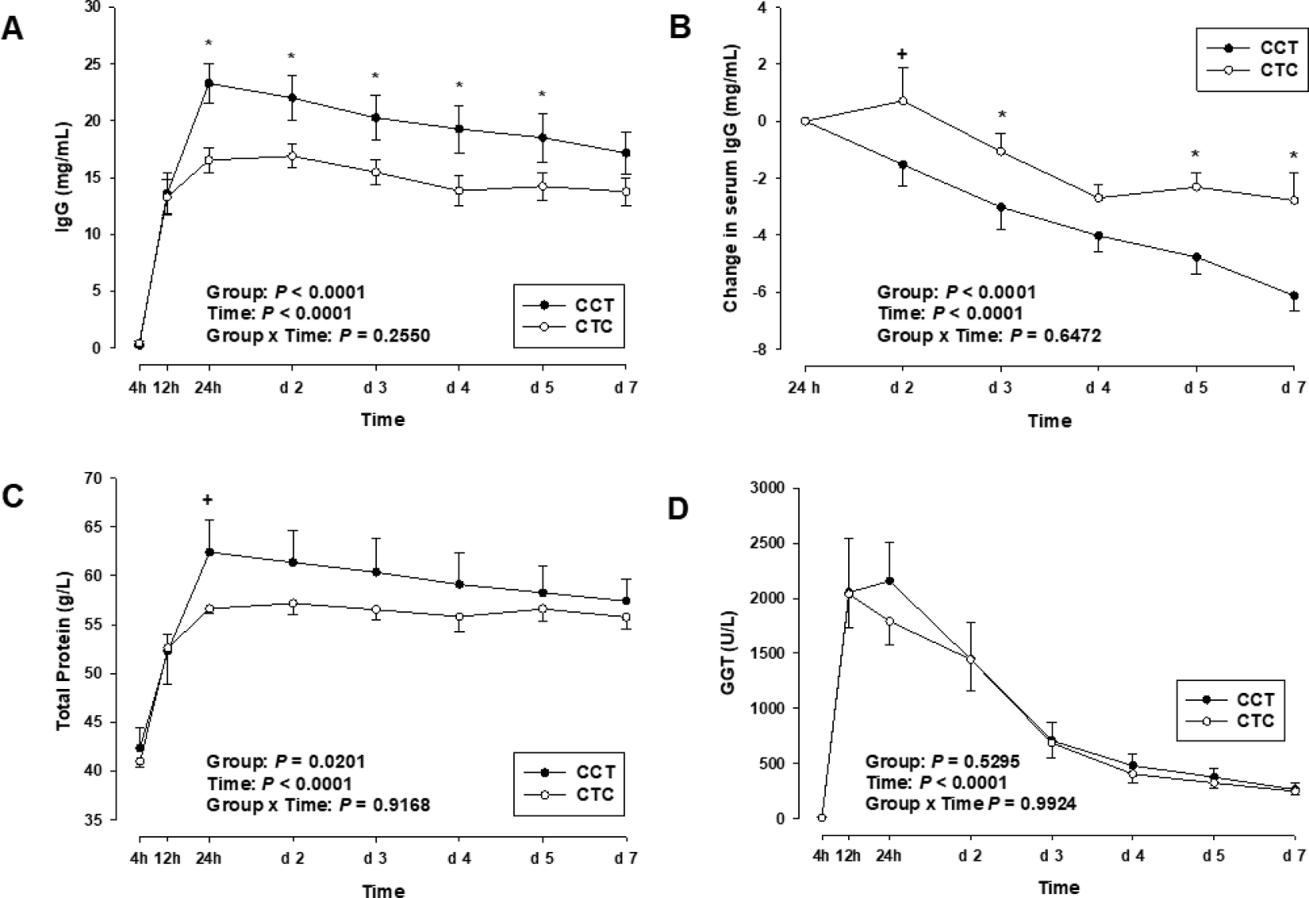
Serum IgG concentration (A), change in serum IgG concentration (B), total protein concentration (C), and serum γ-glutamyl transferase (GGT) activity (D) in calves of the CCT group (n = 6; first colostrum feeding at 4 h p.p.; second colostrum feeding at 12 h p.p.; first enriched bulk tank milk feeding at 24 h p.p.) and the CTC group (n = 6; first colostrum feeding at 4 h p.p.; first enriched bulk tank milk feeding at 12 h p.p.; second colostrum feeding at 24 h p.p.) during the first week of life. Data are presented as mean values ± SEM. Significant differences (*P* < 0.05) between groups are indicated by *, and tendencies toward significance (0.05 ≤ *P* < 0.10) are marked by +.

**Table 1:** Mean values ± SEM of apparent efficiency of absorption (AEA) at 12 h, 24 h, d 2, and d 3 postpartum (p.p.) in CCT (n = 6) and CTC calves (n = 6)

Time p.p.	AEA		*P*-value
	CCT	CTC	
12 h	19.65 (±2.74)	19.02 (±2.20)	0.79
24 h	16.76 (±1.34)	21.96 (±1.45)	0.04
d 2	14.94 (±1.72)	11.70 (±0.76)	0.29
d 3	13.53 (±1.33)	10.46 (±0.77)	0.21

Serum TP concentration was greater in CCT compared with CTC during the experimental period (*P* < 0.05; Figure 1C). In both groups, a significant increase was observed following the first colostrum feeding (*P* < 0.01). The rise of TP concentration continued after the second colostrum feeding in CCT, reaching a peak 24 h p.p. (62.4 ± 3.3 g/L; Figure 1C), which tended to be greater compared with CTC receiving enriched bulk tank milk as a second meal (*P* = 0.07). In contrast, CTC showed no further increase beyond the initial rise observed up to 12 h p.p. Moreover, TP concentration in CTC did not change following the colostrum replacer feeding at 24 h p.p. Total protein concentration in CCT group declined significantly (*P* < 0.05) until d 7 p.p., whereas TP concentration in CTC remained constant from d 2 to 7 (Figure 1C). The GGT activity was not affected by group (*P* = 0.53; Figure 1D). Before the first colostrum feeding, GGT activity was close to detection limit in both groups. Following colostrum intake, serum GGT activity increased significantly in both groups (*P* < 0.01), reaching its peak at 12 h p.p. in CTC, whereas GGT activity in CCT peaked at 24 h p.p. (Figure 1D). Colostrum replacer feeding at 24 h p.p. did not alter GGT activity in CTC. In both groups, GGT activity declined significantly until d 3 p.p. (*P* < 0.01) and remained unchanged thereafter until d 7 p.p.

The objective of the present study was to investigate the impact of an elevated IgG supply at the time of the closing gut barrier on IgG absorption. One of our hypotheses was that high concentrations of IgG in colostrum could partially compensate for the markedly reduced absorptive capacity of the neonatal intestine at 24 h p.p. The average IgG concentrations in colostrum are reported to be 48.2 to 70.0 g/L (Pritchett et al., 1991; Quigley et al., 1994). In comparison, a colostrum replacer with 100 g/L colostral IgG was fed in our study. A study by Matte et al. (1982) showed that calves fed colostrum as late as 48 h p.p. were still able to absorb IgG, supporting the fact that the nonselective absorptive capacity of the intestine is modifiable and does not abruptly cease at 24 h p.p. Transfer of passive immunity is commonly assessed by measuring serum IgG concentrations between 24 and 48 h p.p. (Lopez and Heinrichs, 2022). Calves that fail to receive or absorb sufficient immunoglobulins are classified as having experienced FTPI (Besser et al., 1991; Lombard et al., 2020). In contrast, calves with adequate serum IgG concentrations >10 mg/mL by this time are assumed to have achieved successful passive immunity (**SPI**; Swan et al., 2007; Godden et al., 2019). According to a classification by Lombard et al. (2020), serum IgG concentrations >10 mg/mL are considered fair, whereas concentrations >18 mg/mL are considered good. Based on this classification, serum IgG levels in calves of the CCT group in the present study can be considered good, whereas those in CTC are classified as fair. Importantly, both the CCT and CTC groups achieved SPI already at 24 h p.p.; however, IgG concentrations in neither group reached the “excellent” category, which Lombard et al. (2020) defined at serum concentration >25 mg/mL IgG. Following the second colostrum feeding, serum IgG concentrations were significantly (*P* < 0.05) higher in the CCT group compared with the CTC group, which had already received enriched bulk tank milk at this time. In addition to the 2 colostrum feedings, we provided enriched bulk tank milk during the first 3 d of life, based on evidence indicating that its higher content of bioactive compounds, immunoglobulins, and nutrients supports gastrointestinal maturation in neonatal calves (Van Soest et al., 2022). Although both experimental groups reached their IgG peaks after the second feeding, the increase in serum IgG concentration in the CCT group was markedly higher, rising by 83.2% after the second colostrum intake at 12 h p.p., whereas the CTC group exhibited only a 4.2% increase at 24 h p.p. These findings support our hypothesis that intestinal closure is not fully completed by 24 h of age, allowing for some continued IgG absorption. However, the findings also clearly demonstrate that the absorptive capacity of the neonatal intestine is already substantially reduced at this time. However, in the CTC group, blood sampling on d 2 was conducted within a time frame of 32 to 38 h p.p., which may have missed potential IgG absorption peaks occurring shortly after the second colostrum feeding at 24 h p.p.; thus, the observed increase in serum IgG could underestimate the actual absorptive response in this group. In contrast, previous studies (Hadorn and Blum, 1997) reported an increase in serum IgG after colostrum feeding at 24 h, which may be attributable to more frequent sampling within the first 48 h p.p.

Our findings are further supported by the AEA results. In the CCT group, the AEA of 16.76% was calculated at 24 h p.p., reflecting absorption following the second colostrum feeding at 12 h p.p., whereas in the CTC group, the AEA of 11.7% was calculated on d 2, following the second colostrum feeding at 24 h p.p. This comparison highlights the influence of the timing of the second colostrum administration on IgG absorption, consistent with previous reports of declining AEA with increasing age at first colostrum feeding (Quigley and Drewry, 1998; Jaster, 2005). According to Morin et al. (1997), AEA is not only determined by the timing of colostrum feeding but is also influenced by factors such as the IgG concentration in the colostrum. Average AEA values are generally reported to range between 20% and 35% (Quigley and Drewry, 1998; Lopez and Heinrichs, 2022), which neither of our groups achieved despite both reaching thresholds consistent with SPI. Conneely et al. (2014) investigated the effects of varying volumes of colostrum fed to neonatal calves. Interestingly, calves fed colostrum volumes equal to 10% of their BW showed lower serum IgG concentrations than those fed 8.5% of BW, despite receiving absolutely more IgG. In contrast Morin et al. (1997) and Jaster (2005) concluded that high IgG concentrations in colostrum are consistently associated with higher serum IgG levels in calves. We therefore assume that the relatively large colostrum volume administered (2 × 2.5 L) in our study, combined with the high IgG concentration, may have negatively affected AEA. Accordingly, AEA in this context may be less indicative of absorptive function alone and should be interpreted with caution.

Results on TP followed a similar pattern to that of serum IgG, which can be explained by the fact that a substantial portion of total serum protein in the immediate postnatal phase is composed of immunoglobulins (Barrington et al., 2001). For this reason, TP is conveniently used as an indirect indicator of serum IgG concentration (Naylor and Kronfeld, 1977; Quigley et al., 2002). Similar to IgG, TP levels peaked after the second colostrum feeding in both groups. However, no significant increase was observed in the CTC group, again indicating a limited capacity for protein absorption at 24 h p.p. As already noted for IgG, blood sampling in the CTC group was conducted between 32 and 38 h p.p., which may have missed transient increases in TP shortly after the second colostrum feeding at 24 h p.p. Gamma-glutamyl transferase, an enzyme produced by the ductal epithelial cells of the mammary gland (Weaver et al., 2000), shows a high activity in colostrum and has also been suggested as a marker for IgG absorption (Hadorn and Blum, 1997). As reported by several authors (Thompson and Pauli, 1981; Hammon and Blum, 1998; Ganz et al., 2021), GGT activity in the serum rises significantly after colostrum intake. However, in contrast to IgG and TP, GGT activity plateaued after the first colostrum feeding in both groups. The reasons for this stagnation remain unclear and as already noted for IgG and TP, the timing of blood sampling may have missed transient changes, or rapid metabolism and clearance of GGT may have contributed to the observed plateau. It is also possible that the absorption of GGT is already significantly reduced by 12 h p.p., or alternatively, that metabolic turnover or utilization of GGT increases sharply after the initial uptake. According to Weaver et al. (2000), GGT activities of approximately 200 U/L on the first day and 100 U/L on the fourth day p.p. are indicative of SPI, which was achieved by both groups in our study. However, the marked differences in IgG serum concentrations between the 2 groups over the first 5 d were not reflected in GGT activity data.

Interestingly, although the CCT group exhibited significantly higher absolute serum IgG concentrations, the apparent persistence of IgG in the serum was significantly greater in the CTC group (*P* < 0.05). During the first week of life, serum IgG levels in the CCT group declined by 6.1 ± 0.5 mg/mL, whereas the CTC group experienced a decrease of only 2.8 ± 1.0 mg/mL. This reduction was particularly evident in the CCT group between d 4 and 7, whereas IgG level in CTC remained relatively constant. Hare et al. (2020) reported that apparent IgG persistence was higher in calves fed colostrum or a 1:1 colostrum–whole milk mixture than in calves fed whole milk. However, no significant differences were observed between the colostrum and mixture groups. These results highlight the importance of adequate IgG intake for calf immunity. In contrast, our findings suggest that calves with lower initial serum IgG concentrations may show greater apparent IgG persistence, a phenomenon that is not yet well understood. The postabsorptive gentle decline in IgG concentrations following their peak at 24 h p.p. has been well documented in literature (Mero et al., 1997; Wilm et al., 2018; Lopez et al., 2020). Estimates for the half-life of serum IgG in neonatal calves vary. Murphy et al. (2014) reported a value of 20 d, whereas Besser et al. (1988) found approximately 18 d. Furthermore, Murphy et al. (2014) showed a half-life of 28.5 d for calves fed natural colostrum and only 19.1 d for those receiving colostrum replacer. It is plausible that IgG undergoes catabolism or utilization even during the first week of life. In a study by Yang et al. (2015), an intravenous infusion of IgG resulted in a rapid decline in serum IgG concentrations, explained by the redistribution of IgG molecules into peripheral compartments such as lymph or extracellular fluids. Since our measurements were limited to serum IgG levels within the vascular system, redistribution could plausibly explain the observed postpeak decline, but not necessarily the differential persistence between the 2 feeding groups. FcRn receptors are not only considered key mediators in the intestinal absorption of IgG (Mayer et al., 2002) but are also recognized for their role in extending the persistence of IgG in the circulation (Chaudhury et al., 2003; Jensen et al., 2017). Kacskovics et al. (2006) demonstrated FcRn expression in various epithelial tissues and showed that transchromosomic calves actively protect circulating human IgG from degradation via FcRn. Whether such mechanisms are active in non-transchromosomic calves, and whether large-volume, high-concentration colostrum feedings could saturate FcRn systems in neonatal calves, remains to be elucidated. Additional factors may include the early onset of endogenous IgG synthesis. Jeffcott (1974) described that colostrum-deprived foals initiate IgG production earlier, likely due to the absence of passive immunity. The onset of endogenous IgG synthesis in calves is reported to occur between 36 h and 3 wk of age (Devery et al., 1979; Barrington and Parish, 2001). Whether the relatively stable IgG concentrations observed in the CTC group are attributable to early synthesis or regulated distribution remains speculative. The observed differences in IgG persistence between treatment groups warrants further investigation. In conclusion, our study confirms that early, high-quality colostrum feeding is essential for ensuring adequate passive transfer immunity in neonatal calves. Administration of a high-IgG colostrum replacer at 24 h p.p. did not increase circulating IgG compared with the standard 2-feed regimen, although sampling between 32 and 38 h p.p. may have missed transient absorption peaks. Lower initial IgG in the CTC group was associated with apparently prolonged IgG persistence, but absolute concentrations remained higher in the CCT group, emphasizing that timely colostrum feeding is crucial for optimal calf health. Future research should investigate how dose, timing, and neonatal intestinal absorption interact to shape early immune development.
